# Dosimetric analysis of the alopecia preventing effect of hippocampus sparing whole brain radiation therapy

**DOI:** 10.1186/s13014-015-0555-9

**Published:** 2015-11-26

**Authors:** Anand Mahadevan, Carrie Sampson, Salvatore LaRosa, Scott R. Floyd, Eric T. Wong, Erik J. Uhlmann, Soma Sengupta, Ekkehard M. Kasper

**Affiliations:** Department of Radiation Oncology, Beth Israel Deaconess Medical Center and Harvard Medical School, 330 Brookline Avenue, Boston, MA 02215 USA; Department of Neuro-Oncology, Beth Israel Deaconess Medical Center and Harvard Medical School, Boston, MA USA; Department of Neurosurgery, Beth Israel Deaconess Medical Center and Harvard Medical School, Boston, MA USA

**Keywords:** Hippocampus sparing, Alopecia, IMRT

## Abstract

**Background:**

Whole brain radiation therapy (WBRT) is widely used for the treatment of brain metastases. Cognitive decline and alopecia are recognized adverse effects of WBRT. Recently hippocampus sparing whole brain radiation therapy (HS-WBRT) has been shown to reduce the incidence of memory loss. In this study, we found that multi-field intensity modulated radiation therapy (IMRT), with strict constraints to the brain parenchyma and to the hippocampus, reduces follicular scalp dose and prevents alopecia.

**Methods:**

Suitable patients befitting the inclusion criteria of the RTOG 0933 trial received Hippocampus sparing whole brain radiation. On follow up, they were noticed to have full scalp hair preservation. 5 mm thickness of follicle bearing scalp in the radiation field was outlined in the planning CT scans. Conventional opposed lateral WBRT radiation fields were applied to these patient-specific image sets and planned with the same nominal dose of 30 Gy in 10 fractions. The mean and maximum dose to follicle bearing skin and Dose Volume Histogram (DVH) data were analyzed for conventional and HS-WBRT. Paired *t*-test was used to compare the means.

**Results:**

All six patients had fully preserved scalp hair and remained clinically cognitively intact 1–3 months after HS-WBRT. Compared to conventional WBRT, in addition to the intended sparing of the Hippocampus, HS-WBRT delivered significantly lower mean dose (22.42 cGy vs. 16.33 cGy, *p* < 0.0001), V_24_ (9 cc vs. 44 cc, *p* < 0.0000) and V_30_ (9 cc vs. 0.096 cc, *p* = 0.0106) to follicle hair bearing scalp and prevented alopecia. There were no recurrences in the Hippocampus area.

**Conclusions:**

HS-WBRT, with an 11-field set up as described, while attempting to conserve hippocampus radiation and maintain radiation dose to brain inadvertently spares follicle-bearing scalp and prevents alopecia.

## Background

Cognitive impairment after WBRT is a significant problem with 50–90 % of patients showing measurable decline on testing and 10–15 % showing progressive clinical cognitive decline, with significant effect on quality of life [1–4]. While medical therapies (donapezil and memantine) have been used to prevent and treat cognitive decline associated with WBRT, they can be costly and may result in adverse effects or non-compliance [5, 6]. HS-WBRT has shown to be safe, feasible and promising in reducing cognitive decline [7–9].

HS-WBRT, usually performed with intensity modulation (IMRT), utilizes iterative planning to restrict dose to hippocampus while maintaining dose to the rest of the brain parenchyma as anatomically defined. The strict dose-volume parameters have been studied and prescribed in the recently completed and published RTOG trail, which showed significantly less decline in cognitive function with HS-WBRT [7].

Radiation induced alopecia is well-known and significant side effect of conventional WBRT [10, 11]. While the significance of radiation-induced alopecia has been recognized, and attempts been made to mitigate or prevent it, it has been accepted as an unavoidable consequence. Dose response to alopecia [12] and other interventions to mitigate alopecia have been described. We report dose volume analysis to scalp, comparing conventional and HS-WBRT. This was in response to noticing hair preservation in all our patients treated with an 11-field HS-WBRT.

## Materials and Methods

**Fig. 1:**
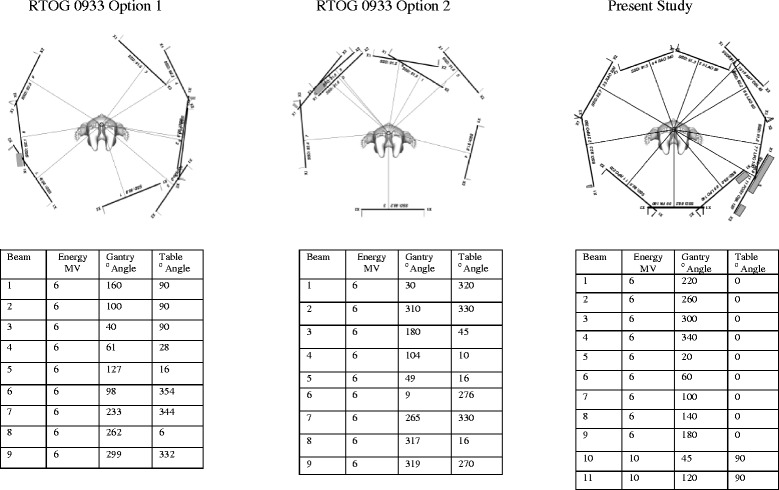
Field setup used in RTOG 0933 and in current study

**Fig. 2:**
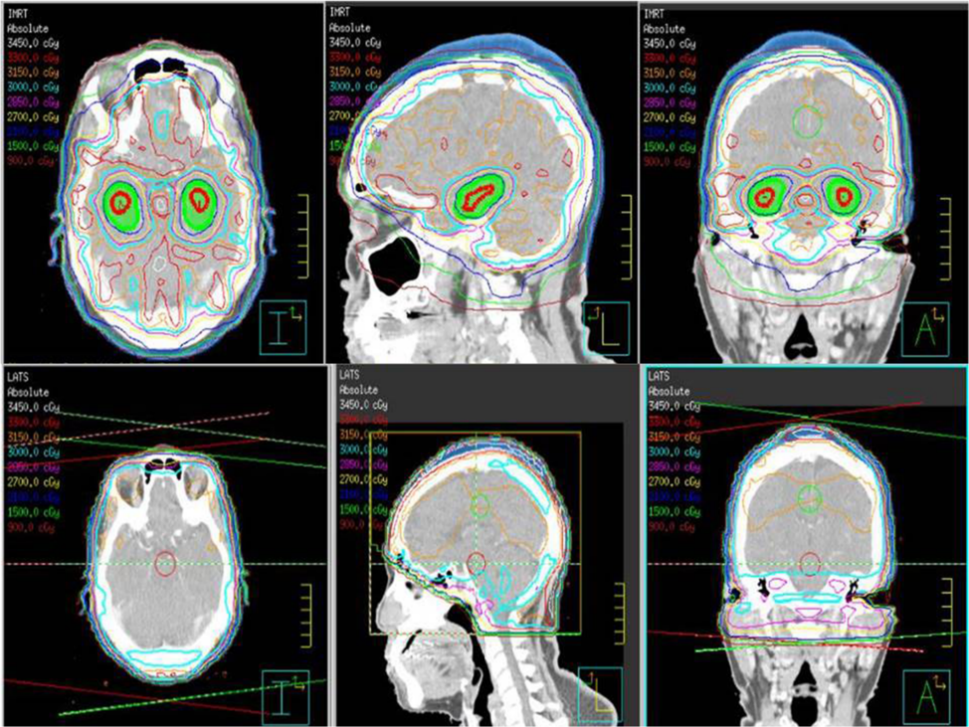
Graphic Plan of HS-WBRT (*top 3 panels*) and Conventional WBRT (*bottom 3 panels*)

Patients with brain metastasis who fulfill the eligibility criteria for RTOG 0933 were included in this study. No patients had preexisting alopecia, including systemic chemotherapy induced alopecia, and had a full complement of scalp hair. The first 6 consecutive patients who received HS-WBRT who were noticed to have full preservation of scalp hair on follow up were analyzed. All patients had diagnostic brain MRI with contrast. A thermoplastic mask immobilization was used with CT simulation. CT-MRI fusion was used for target volume delineation. A simplified and reproducible in-house 11-field IMRT plan, with 9 coplanar (0° couch angle) of 6MV photons and 2 coronal (90° couch angle) beams of 10MV photons were used. This was preferred to the recommended beam arrangements in the RTOG trial, which involved multiple gantry and couch angles. The suggested field setup in the RTOG trial and the setup used in this study are compared in Fig. 1. All dosimetric guidelines and parameters used in the RTOG 0933 trial were used and met. The dose volume constraints to the hippocampus, the hippocampus avoidance zone, brain parenchyma and the organs at risk – including the lenses, optic nerves and optic chiasm, were met for all patients. A representative treatment plan is shown in Fig. 2.

**Fig. 3:**
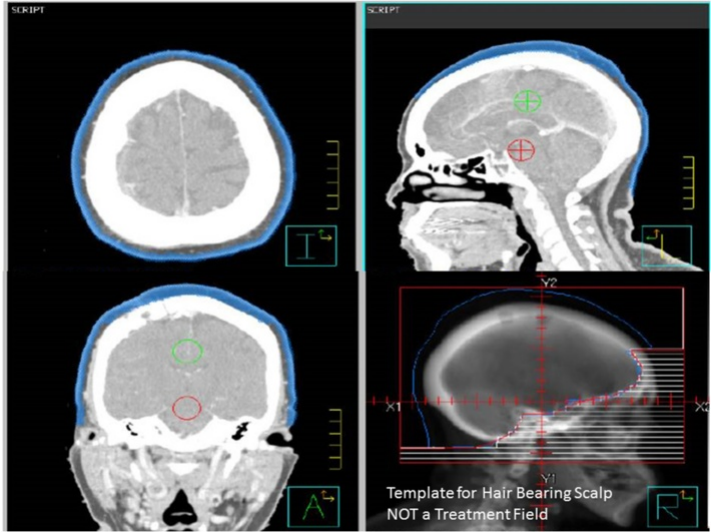
Auotcontour of Hair Follicle bearing scalp. *Lower Right panel* is NOT a treatment field but a template for the extent of temporal hair bearing scalp

5 mm hair follicle bearing scalp was auto contoured in all CT simulation image data sets retrospectively (Fig. 3). Conventional opposed lateral fields with MLC (Multi Leaf Collimator) blocks were applied to all image sets as would be used to treat with conventional WBRT with 6MV photons. The dose volume parameters to the follicle-bearing scalp for both plans were calculated.

All patients were followed one month after and three monthly thereafter until progression or death, with clinical examination including mini mental state and brain MRI. As these patients were treated outside protocol, a complete neuro-cognitive assessment battery of tests was not performed.

Paired *t*-test to compare the means was utilized using STATA^R^ software (Statacorp LP, College Station, Texas 77845, USA).

The retrospective review was IRB approved – hence the protocol for the research project has been approved by a suitably constituted Ethics Committee of the institution within which the work was undertaken and that it conforms to the provisions of the Declaration of Helsinki, and the study does not violate the policies and/or procedures established by the Journal.

## Results

### Clinical outcomes

**Fig. 4:**
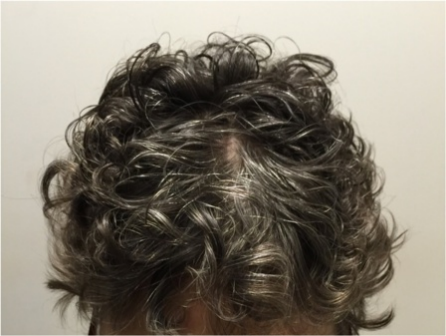
Hair preservation 1 and 3 months after HS-WBRT

At the time of their one-month and last follow up all patients had full scalp hair preservation (Fig. 4), by their own subjective reporting and on clinical examination. All patients experience fatigue, but no other neurological toxicity. One patient died of a stroke, presumed unrelated, one month after HS-WBRT. Two patients had recurrent brain metastasis. No patients failed in the Hippocampus avoidance zone or in the skull base. All six patients had clinically preserved cognitive function.

### Dosimetric outcomes

**Fig. 5:**
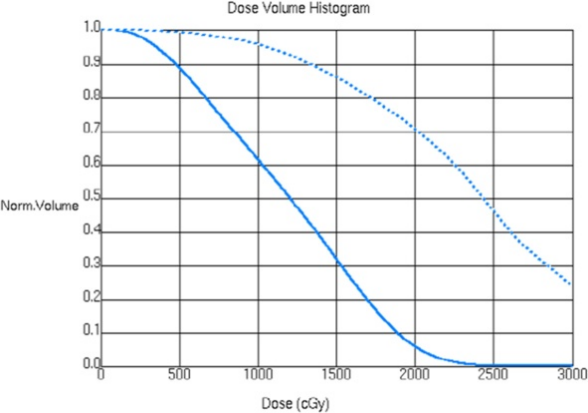
DVH (Dose Volume Histogram) showing scalp dose with conventional WBRT (dashed line) and HS-WBRT (solid line)

The mean, maximum, V24 (volume of follicle bearing scalp receiving 24 Gy) and V30 (volume of follicle bearing scalp receiving 30 Gy) for each patient are shown in Table 1. Compared to HS-WBRT, conventional WBRT delivered significantly higher mean dose to follicle bearing scalp (16.33 cGy vs. 22.42 cGy, *p* < 0.0001). The volume of radiated hair bearing scalp receiving 24 Gy – V_24_ (9 cc vs. 44 ml, *p* < 0.0000) and V_30_ (0.096 ml vs. 9 ml), *p* = 0.0106) were also significantly higher. Dose Volume Histograms (DVHs) illustrating differences in the volume of hair follicle bearing scalp-receiving threshold dose for alopecia (V_24_) and prescribed dose (V_30_) are shown in Fig. 5.

**Table 1:** Hair follicle bearing scalp dose in cGy

Patient	Mean		Max		V_24_		V_30_	
	LATS	HS-WBRT	LATS	HS-WBRT	LATS	Hs-WBRT	LATS	HS-WBRT
1	2364.8	1189.7	3586.7	2997.8	51.94	0.26	23.71	0
2	2247.7	1715.6	3375	3795.5	45.59	11.5	6.84	0.21
3	2282.1	1623.9	3383.2	3107.8	46.2	6.47	6.77	0.01
4	2171.2	1827	3333.7	3280.6	40.16	16.69	3.94	0.21
5	2313.2	1646.7	3418	3352.1	50.59	10.56	11.62	0.08
6	2076	1797.4	3205.8	3238.7	30.33	13.38	1.17	0.07
Mean	2242.5	1633.383	3383.733	3295.417	44.135	9.81^a^	9.008333	0.096667^a^

*LATS* Opposed lateral fields, *HS-WBRT* Hippocampus sparing – whole brain radiation therapy, *Max* Maximum scalp dose, *V*_*24*_ Volume of hair bearing skin receiving 24 Gyin cc, *V*_*30*_ Volume of hair bearing skin receiving 30 Gyin cc.

^a^Statistically significant

## Discusssion

Radiation to scalp with sufficient doses can lead to alopecia [12]. Conventional WBRT does not seek to limit dose to scalp and causes alopecia. Hippocampus sparing WBRT while attempting to rigidly enforce dose compliance to brain and normal structures including the hippocampus and the hippocampus avoidance zone, can spare hair follicle bearing scalp of significant radiation and prevents alopecia.

WBRT has long been the standard of care for brain metastasis. It significantly improves survival when indicated for prophylaxis [13, 14] and therapy [15]. Neurocognitive decline after WBRT is a recognized complication [16–18]. Attempts have been made to mitigate this with adjuvant medication modulating neurotransmission [5, 6]. Medication to prevent cognitive decline after WBRT can be costly, have problems with side effects and compliance and the benefits are modest. Randomized trials have also shown that addition of WBRT to local therapy (Stereotactic radiosurgery or resection) may not improve survival but significantly decreases the likelihood of distant brain failure [3, 4, 19, 20]. Hence there is a trend to omit WBRT altogether to avoid cognitive decline. However recurrent brain metastasis, which could have been prevented, can also be detrimental neurologically and may cause cognitive decline [21, 22]. With the realization of sensitive structures of the brain involved in neuro-cognitive processing in the Hippocampus area [9], and the likelihood of harboring metastasis or recurrence of metastasis in this zone being rare [8, 23], HS-WBRT has emerged as a viable treatment option to preserve the benefits of WBRT and yet decrease the probability of neuro-cognitive decline. Indeed a recently completed co-operative group trial confirmed the feasibility and the neuro-cognitive protective function of HS-WBRT [7]. This strategy avoids the additional cost, side effects and frequent non-compliance associated with the medications for preventing and treating neuro-cognitive decline. Encouraged by this our group initiated HS-WBRT for patients meeting the eligibility in this trial.

HS-WBRT has been shown to be feasible by multiple groups, with or without integrated boost using linear accelerator-based multi-field IMRT [24], Volumetric Modulated Arc Therapy (VMAT) [25–27] or Tomotherapy techniques [28]. Specific IMRT techniques have been recommended and used in the RTOG 0933 trial [24]. However the 9-Field IMRT technique recommended in this trial with multiple individual couch angles ranging from 6–354° and multiple individual gantry angles from 9–319° were found, by us, to be unwieldy, cumbersome, time consuming and fraught with potential collision and setup errors. After experimenting with multiple simple beam arrangements we found it feasible to reproducibly achieve all DVH constraints as prescribed, with two fixed standard couch position in 0° and 90° and fixed coplanar gantry angles, thereby eliminating collision risk and significantly simplifying and shortening of patients’ setup.

Tolerance dose to scalp has been reported for permanent alopecia after definitive radiation therapy for brain tumors [12, 29]. Hair loss, at least temporarily, occurs in most patients receiving WBRT with conventional fractionation [11]. This has been consistently shown to translate to poor quality of life within their reduced life expectancy in these patients [10, 14, 30, 31]. Attempts have been made to use topical nitroxides [32, 33], prostagladin E [34], botulinum toxin [35] and vasoconstrictors [36] to decrease the incidence and severity of alopecia after radiation therapy. However the no clinical benefit was found. Scalp dose-limiting WBRT techniques have been described previously. Roberge et al. measured surface scalp doses with TLD and calculated doses at 5 mm thickness and reported decrease in mean doses of 53 % and 38 % respectively [37]. Mancini et al. similarly demonstrated decrease scalp dose after VMAT- IMRT [38]. In a more detailed analysis De Puysseleyr et al., using preliminary cadaveric data and in a prospective study using VMAT IMRT for scalp sparing, showed 37 % reduction to the median dose to the hair follicle volume [39]. However, this unfortunately did not translate into clinical benefit with poor QOL and Alopecia scores. Differences in technique (VMAT vs. Tomotherapy vs. Multi Field IMRT), energy, beam arrangement, objectives (hippocampus sparing vs. scalp sparing), contouring of hair bearing scalp, DVH constraints and DVH parameters could account for the apparent differences in these outcomes.

## Conclusion

While other IMRT set-ups have independently shown hippocampus sparing or scalp sparing, in our study we demonstrate dual benefit of Hippocampus sparing and, unexpectedly, scalp sparing effects of an 11-field IMRT technique. This is likely to even more enhance the quality of life in suitable patients receiving WBRT for brain metastasis, and will be worthy of evaluating prospectively.

## Abbreviations

HS-WBRT: hippocampus sparing WBRT
IMRT: intensity modulated radiation therapy
WBRT: whole brain radiation therapy

## References

[1] Taphoorn MJ, Klein M (2004). Cognitive deficits in adult patients with brain tumours. Lancet Neurol.

[2] Brown PD (2015). NCCTG N0574 (Alliance): A phase III randomized trial of whole brain radiation therapy (WBRT) in addition to radiosurgery (SRS) in patients with 1 to 3 brain metastases. J Clin Oncol.

[3] Kocher M, Soffietti R, Abacioglu U, Villa S, Fauchon F, Baumert BG (2011). Adjuvant whole-brain radiotherapy versus observation after radiosurgery or surgical resection of one to three cerebral metastases: results of the EORTC 22952–26001 study. J Clin Oncol.

[4] Chang EL, Wefel JS, Hess KR, Allen PK, Lang FF, Kornguth DG (2009). Neurocognition in patients with brain metastases treated with radiosurgery or radiosurgery plus whole-brain irradiation: a randomised controlled trial. Lancet Oncol.

[5] Brown PD, Pugh S, Laack NN, Wefel JS, Khuntia D, Meyers C (2013). Memantine for the prevention of cognitive dysfunction in patients receiving whole-brain radiotherapy: a randomized, double-blind, placebo-controlled trial. Neuro-Oncol.

[6] Shaw EG, Rosdhal R, D’Agostino RB, Lovato J, Naughton MJ, Robbins ME (2006). Phase II study of donepezil in irradiated brain tumor patients: effect on cognitive function, mood, and quality of life. J Clin Oncol.

[7] Gondi V, Pugh SL, Tome WA, Caine C, Corn B, Kanner A (2014). Preservation of memory with conformal avoidance of the hippocampal neural stem-cell compartment during whole-brain radiotherapy for brain metastases (RTOG 0933): a phase II multi-institutional trial. J Clin Oncol.

[8] Gondi V, Tome WA, Marsh J, Struck A, Ghia A, Turian JV (2010). Estimated risk of perihippocampal disease progression after hippocampal avoidance during whole-brain radiotherapy: safety profile for RTOG 0933. Radiother Oncol.

[9] Gondi V, Tome WA, Mehta MP (2010). Why avoid the hippocampus? A comprehensive review. Radiother Oncol.

[10] Steinmann D, Paelecke-Habermann Y, Geinitz H, Aschoff R, Bayerl A, Bolling T (2012). Prospective evaluation of quality of life effects in patients undergoing palliative radiotherapy for brain metastases. BMC Cancer.

[11] Gerrard GE, Prestwich RJ, Edwards A, Russon LJ, Richards F, Johnston CF (2003). Investigating the palliative efficacy of whole-brain radiotherapy for patients with multiple-brain metastases and poor prognostic features. Clin Oncol.

[12] Lawenda BD, Gagne HM, Gierga DP, Niemierko A, Wong WM, Tarbell NJ (2004). Permanent alopecia after cranial irradiation: dose–response relationship. Int J Radiat Oncol Biol Phys.

[13] Auperin A, Arriagada R, Pignon JP, Le Pechoux C, Gregor A, Stephens RJ (1999). Prophylactic cranial irradiation for patients with small-cell lung cancer in complete remission. Prophylactic Cranial Irradiation Overview Collaborative Group. N Engl J Med.

[14] Slotman BJ, Mauer ME, Bottomley A, Faivre-Finn C, Kramer GW, Rankin EM (2009). Prophylactic cranial irradiation in extensive disease small-cell lung cancer: short-term health-related quality of life and patient reported symptoms: results of an international Phase III randomized controlled trial by the EORTC Radiation Oncology and Lung Cancer Groups. J Clin Oncol.

[15] Horton J, Baxter DH, Olson KB (1971). The management of metastases to the brain by irradiation and corticosteroids. Am J Roentgenol.

[16] Soffietti R, Kocher M, Abacioglu UM, Villa S, Fauchon F, Baumert BG (2013). A European Organisation for Research and Treatment of Cancer phase III trial of adjuvant whole-brain radiotherapy versus observation in patients with one to three brain metastases from solid tumors after surgical resection or radiosurgery: quality-of-life results. J Clin Oncol.

[17] Le Pechoux C, Laplanche A, Faivre-Finn C, Ciuleanu T, Wanders R, Lerouge D (2011). Clinical neurological outcome and quality of life among patients with limited small-cell cancer treated with two different doses of prophylactic cranial irradiation in the intergroup phase III trial (PCI99-01, EORTC 22003–08004, RTOG 0212 and IFCT 99–01). Ann Oncol.

[18] Sun A, Bae K, Gore EM, Movsas B, Wong SJ, Meyers CA (2011). Phase III trial of prophylactic cranial irradiation compared with observation in patients with locally advanced non-small-cell lung cancer: neurocognitive and quality-of-life analysis. J Clin Oncol.

[19] Aoyama H, Shirato H, Tago M, Nakagawa K, Toyoda T, Hatano K (2006). Stereotactic radiosurgery plus whole-brain radiation therapy vs stereotactic radiosurgery alone for treatment of brain metastases: a randomized controlled trial. Jama.

[20] Patchell RA, Tibbs PA, Regine WF, Dempsey RJ, Mohiuddin M, Kryscio RJ (1998). Postoperative radiotherapy in the treatment of single metastases to the brain: a randomized trial. Jama.

[21] Aoyama H, Tago M, Kato N, Toyoda T, Kenjyo M, Hirota S (2007). Neurocognitive function of patients with brain metastasis who received either whole brain radiotherapy plus stereotactic radiosurgery or radiosurgery alone. Int J Radiat Oncol Biol Phys.

[22] Meyers CA, Smith JA, Bezjak A, Mehta MP, Liebmann J, Illidge T (2004). Neurocognitive function and progression in patients with brain metastases treated with whole-brain radiation and motexafin gadolinium: results of a randomized phase III trial. J Clin Oncol.

[23] Ghia A, Tome WA, Thomas S, Cannon G, Khuntia D, Kuo JS (2007). Distribution of brain metastases in relation to the hippocampus: implications for neurocognitive functional preservation. Int J Radiat Oncol Biol Phys.

[24] Gondi V, Tolakanahalli R, Mehta MP, Tewatia D, Rowley H, Kuo JS (2010). Hippocampal-sparing whole-brain radiotherapy: a “how-to” technique using helical tomotherapy and linear accelerator-based intensity-modulated radiotherapy. Int J Radiat Oncol Biol Phys.

[25] Hsu F, Carolan H, Nichol A, Cao F, Nuraney N, Lee R (2010). Whole brain radiotherapy with hippocampal avoidance and simultaneous integrated boost for 1–3 brain metastases: a feasibility study using volumetric modulated arc therapy. Int J Radiat Oncol Biol Phys.

[26] Awad R, Fogarty G, Hong A, Kelly P, Ng D, Santos D (2013). Hippocampal avoidance with volumetric modulated arc therapy in melanoma brain metastases - the first Australian experience. Radiat Oncol.

[27] Oehlke O, Wucherpfennig D, Fels F, Frings L, Egger K, Weyerbrock A, et al. Whole brain irradiation with hippocampal sparing and dose escalation on multiple brain metastases : Local tumour control and survival. Strahlentherapie und Onkologie : Organ der Deutschen Rontgengesellschaft [et al.]. 2015. doi:10.1007/s00066-014-0808-9. PubMed 10.1007/s00066-014-0808-9 25592907

[28] Kim KH, Cho BC, Lee CG, Kim HR, Suh YG, Kim JW (2015). Hippocampus-sparing whole-brain radiotherapy and simultaneous integrated boost for multiple brain metastases from lung adenocarcinoma: early response and dosimetric evaluation. Technol Cancer Res Treat.

[29] Haider M, Hamadah I, Almutawa A (2013). Radiation- and chemotherapy-induced permanent alopecia: case series. J Cutan Med Surg.

[30] Bottomley A, Flechtner H, Efficace F, Vanvoorden V, Coens C, Therasse P (2005). Health related quality of life outcomes in cancer clinical trials. Eur J Cancer.

[31] Steinmann D, Schafer C, van Oorschot B, Wypior HJ, Bruns F, Bolling T (2009). Effects of radiotherapy for brain metastases on quality of life (QoL). Prospective pilot study of the DEGRO QoL working party. Strahlentherapie Onkol.

[32] Metz JM, Smith D, Mick R, Lustig R, Mitchell J, Cherakuri M (2004). A phase I study of topical Tempol for the prevention of alopecia induced by whole brain radiotherapy. Clin Cancer Res.

[33] Hahn SM, Krishna CM, Samuni A, DeGraff W, Cuscela DO, Johnstone P (1994). Potential use of nitroxides in radiation oncology. Cancer Res.

[34] Geng L, Hanson WR, Malkinson FD (1992). Topical or systemic 16, 16 dm prostaglandin E2 or WR-2721 (WR-1065) protects mice from alopecia after fractionated irradiation. Int J Radiat Biol.

[35] Hyun MY, Kim BJ, Lee C, Kim JW (2014). Radiation-induced alopecia treated with Botulinum toxin type a injection. Plast Reconstr Surg Glob Open.

[36] Soref CM, Fahl WE (2015). A new strategy to prevent chemotherapy and radiotherapy-induced alopecia using topically applied vasoconstrictor. Int J Cancer.

[37] Roberge D, Parker W, Niazi TM, Olivares M (2005). Treating the contents and not the container: dosimetric study of hair-sparing whole brain intensity modulated radiation therapy. Technol Cancer Res Treat.

[38] Mancini BR KL, Shaitelman SF, Yan D, Kestin LL, Grills IS (2010). Intensity modulated or volumetric modulated radiation therapy (IMRT or VMAT) to reduce alopecia, xerostomia, and otitis after whole brain radiation therapy for brain metastases: a planning analysis. Int J Radiat Oncol Biol Phys.

[39] De Puysseleyr A, Van De Velde J, Speleers B, Vercauteren T, Goedgebeur A, Van Hoof T (2014). Hair-sparing whole brain radiotherapy with volumetric arc therapy in patients treated for brain metastases: dosimetric and clinical results of a phase II trial. Radiat Oncol.

